# Cortical tethering of mitochondria by the anchor protein Mcp5 enables uniparental inheritance

**DOI:** 10.1083/jcb.201901108

**Published:** 2019-10-03

**Authors:** Leeba Ann Chacko, Kritika Mehta, Vaishnavi Ananthanarayanan

**Affiliations:** Centre for BioSystems Science and Engineering, Indian Institute of Science, Bangalore, India

## Abstract

During sexual reproduction in eukaryotes, processes such as active degradation and dilution of paternal mitochondria ensure maternal mitochondrial inheritance. In the isogamous organism fission yeast, we employed high-resolution fluorescence microscopy to visualize mitochondrial inheritance during meiosis by differentially labeling mitochondria of the two parental cells. Remarkably, mitochondria, and thereby mitochondrial DNA from the parental cells, did not mix upon zygote formation but remained segregated at the poles by attaching to clusters of the anchor protein Mcp5 via its coiled-coil domain. We observed that this tethering of parental mitochondria to the poles results in uniparental inheritance of mitochondria, wherein two of the four spores formed subsequently contained mitochondria from one parent and the other spores contained mitochondria from the other parent. Further, the presence of dynein on an Mcp5 cluster precluded the attachment of mitochondria to the same cluster. Taken together, we reveal a distinct mechanism that achieves uniparental inheritance by segregation of parental mitochondria.

## Abstract (precis)

Paternal mitochondria are removed during eukaryotic sexual reproduction to ensure maternal mitochondrial inheritance. Chacko et al. show that fission yeast uses an anchor protein to physically separate and tether parental mitochondria to the cortex during meiosis, thereby achieving uniparental mitochondrial inheritance.

## Introduction

Mitochondria are cellular organelles responsible for the generation of energy-rich adenosine triphosphate molecules in eukaryotic cells. In addition to this and other important functions, mitochondria carry their own genetic material in the form of mitochondrial DNA (mtDNA) nucleoids. During meiosis, in contrast to the nuclear genome, mitochondrial genes follow a non-Mendelian pattern of segregation through tightly controlled mechanisms that typically favor uniparental inheritance, or the passing down of mitochondria predominantly from a single parent to the progeny. In several eukaryotes, maternal inheritance is the preferred mode of uniparental inheritance. Maternal inheritance is brought about by one of many ways, including subjecting paternal mitochondria to (1) sequestration and exclusion (Yu and Russell, 1992), (2) selective lysosomal degradation via ubiquitination (Sutovsky et al., 1999, 2000), or (3) simple dilution due to the large size of the female gamete in comparison to the male gamete (Birky, 1995; Wilson and Xu, 2012). Uniparental mitochondrial inheritance has been suggested to be important for preventing the propagation of selfish cytoplasmic transposable elements that could affect the nuclear genome (Cosmides and Tooby, 1981; Hoekstra, 2000).

In the unicellular eukaryote budding yeast *Saccharomyces cerevisiae*, mitochondria are biparentally inherited by the meiotic progeny due to mixing of mitochondria from both parental cells upon zygote formation (Thomas and Wilkie, 1968; Strausberg and Perlman, 1978; Zinn et al., 1987). However, mtDNA that occur in the form of nucleoids seemingly remain anchored to their original locations in the zygote, thereby giving rise to homoplasmic cells within a few rounds of vegetative division following sporulation (Nunnari et al., 1997). During mitosis in *S. cerevisiae*, mitochondria in the mother cell are tethered to the cell membrane via the mitochondria–ER cortex anchor (MECA) structure containing the protein Num1 (Heil-Chapdelaine et al., 2000; Klecker et al., 2013; Lackner et al., 2013; Ping et al., 2016). Tethering of mitochondria by Num1 aids in the retention of a mitochondrial population within the mother cell (Lackner et al., 2013), while another population is transported on actin cables to the bud by the activity of the myosin V, Myo2 (Altmann et al., 2008; Förtsch et al., 2011). The Num1 homologue in fission yeast (*Schizosaccharomyces pombe*), Mcp5, is expressed specifically during prophase I of meiosis (Saito et al., 2006; Yamashita and Yamamoto, 2006) and is required for the anchoring and thereby activation of the motor protein cytoplasmic dynein that powers the oscillatory movement of the zygotic horsetail-shaped nucleus (Yamamoto et al., 1999; Tolic et al., 2009; Ananthanarayanan et al., 2013).

Interphase mitochondria in fission yeast remain associated with microtubules, and their fission dynamics are dictated by the dynamics of the underlying microtubules (Yaffe et al., 1996; Chiron et al., 2008; Fu et al., 2011; Mehta et al., 2019). This relationship between microtubules and mitochondria is also essential for independent segregation of mitochondria during mitosis (Mehta et al., 2019). However, it is unclear how mitochondria are segregated among the four spores that result from meiotic cell division in fission yeast. It has been suggested that like *S. cerevisiase*, *S. pombe* also undergoes biparental mitochondrial inheritance in crosses between strains resistant and sensitive to antibiotics (Thrailkill et al., 1980), but direct evidence for this process in wild-type cells has been lacking.

Here, we report that fission yeast cells in fact undergo uniparental mitochondrial inheritance during meiosis due to the tethering of mitochondria to the cortex during the initial stages of meiosis. Our results thus reveal a unique mechanism for facilitating uniparental inheritance that relies on physical segregation of parental mitochondria in a heteroplasmic zygote by the activity of the anchor protein Mcp5.

## Results

### Mitochondria are preferentially localized at the poles of meiotic cells

**Figure 1.**
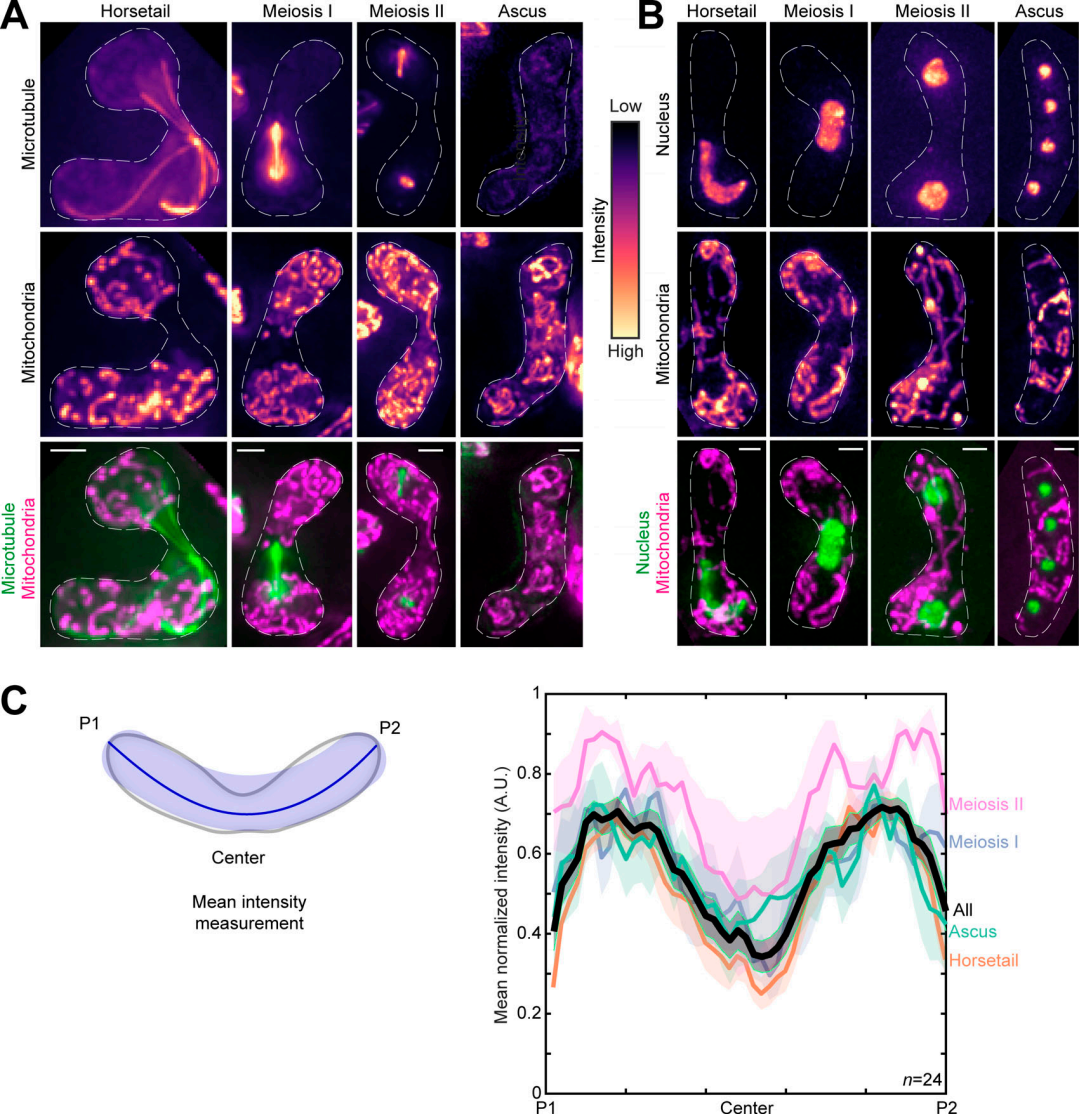
**Mitochondria remain close to the cell poles during meiosis. (A)** Maximum-intensity–projected images of microtubules (top) and mitochondria (middle) represented in the intensity map to the right of the images, and their merge (bottom) during the different stages of meiosis indicated (strain KI001xPT1651; see Table S1). **(B)** Maximum-intensity–projected images of the nucleus (top) and mitochondria (middle) represented in the intensity map to the left of the images, and their merge (bottom) during the different stages of meiosis indicated (strain FY15112; see Table S1). In A and B, scale bars represent 2 µm and dashed lines represent cell outlines. **(C)** Schematic (left) of the mean intensity measurement along the length of a zygote from pole P1, through the center, to pole P2. Plot of mean normalized intensities (right) from different stages of meiosis (colored lines) and their combined mean intensities (black line, *n* = 24) obtained from the data in A. The shaded regions represent the SEM.

To the best of our knowledge, there exists no comprehensive study on the changes of the mitochondrial network upon onset of meiosis in fission yeast. Therefore, we first set out to visualize mitochondria during the fission yeast meiotic cycle. We achieved this by inducing meiosis in parental cells that had fluorescently labeled mitochondria and microtubules (Fig. 1 A and Video 1, top), or mitochondria and nucleus (Fig. 1 B and Video 1, bottom).

Based on the microtubule organization and nuclear morphology, the discernible stages of meiosis were designated as horsetail, meiosis I, meiosis II, and ascus (Cipak et al., 2014). In contrast to interphase mitochondria, during meiosis, mitochondria appeared predominantly fragmented and detached from the microtubules (e.g., Fig. 1 A, horsetail). Further, the mean normalized intensity of mitochondria across the cell for all stages revealed preferential localization of mitochondria to the poles of the cell (Fig. 1 C).

### Parental mitochondria do not mix upon zygote formation

**Figure 2.**
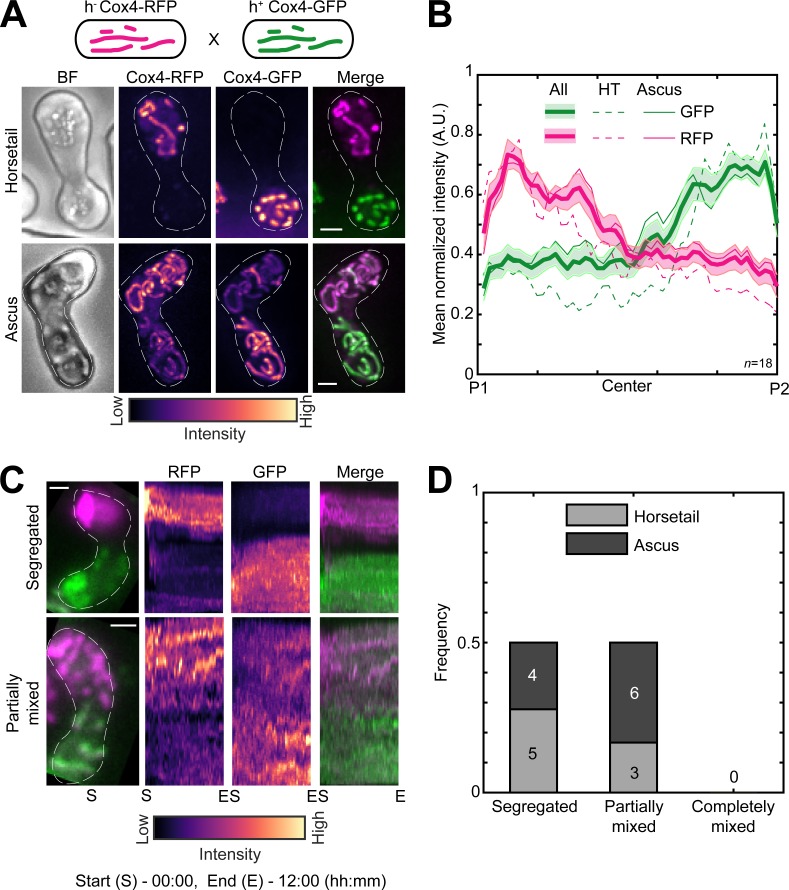
**Parental mitochondria remain segregated upon conjugation. (A)** Schematic of the cross performed (top, strain PT1650xPT1651; see Table S1), maximum-intensity–projected images of bright-field channel (BF; first from left), images of mitochondria labeled with Cox4-RFP (second from left) and Cox4-GFP (third from left) represented in the intensity map to the bottom of the images, and their merge (right) during the early stage (horsetail, top) and late stage (ascus, bottom) of meiosis. **(B)** Plot of mean normalized intensities of RFP (magenta lines) and GFP (green lines) in the horsetail stage (HT; dashed lines, *n =* 8), ascus stage (Ascus; solid lines, *n =* 10), and the stages combined (All; thick solid lines) across the length of the cell from the cross indicated in A (*n* = 18). Shaded regions represent SEM. **(C)** Representative maximum-intensity–projected images (left) and kymographs of time-lapse movies of RFP channel (second from left), GFP channel (third from left), and their merge (right) of meiotic cells resulting from the cross indicated in A, exhibiting the segregated phenotype (top) and partially mixed phenotype (bottom). The intensity map of kymographs of the GFP and RFP channel is indicated to the bottom of the images. S denotes start of imaging at 00:00, and E denotes end of imaging at 12:00 (hours:minutes). **(D)** Stacked bar plot of frequency of segregated, partially mixed and completely mixed phenotypes observed in horsetail (light gray) and ascus (dark gray) stages from the data in B. In A and C, scale bars represent 2 µm and dashed lines represent cell outlines.

Next, we sought to understand how mitochondria are inherited during fission yeast meiosis. To this end, we employed cells of opposite mating types whose mitochondria were labeled with different fluorophores, GFP and RFP. We induced meiosis in these cells and followed the mitochondrial organization during the early horsetail stage and in the final stage, after formation of ascospores. Interestingly, we observed that the differently labeled mitochondria from the parental cells remained predominantly segregated at the poles of the cell and did not undergo mixing in the early stage (Fig. 2 A, top; and Video 2, left). Upon formation of spores within the ascus, mitochondria again remained predominantly unmixed, with two of the spores exhibiting a higher GFP signal and the two other a higher RFP signal (Fig. 2 A, bottom; and Video 2, right). These observations were consistent with our measurement of mean normalized mitochondrial intensities across the length of the cell at both early and late stages (Fig. 2 B). We additionally visualized meiotic mitochondrial inheritance in a cross between a cell containing fluorescently labeled mitochondria and a cell containing unlabeled mitochondria. Here, too, we observed localization of mitochondrial signal to one side of the zygote and two spores of the resulting ascus (Fig. S1, A and B).

In all these experiments, the mitochondrial inner membrane protein Cox4 was used as a fluorescent reporter for the mitochondria. To rule out any effects from differential dynamics of the mitochondrial compartments (Sukhorukov et al., 2010), we used another fluorescent reporter protein for the mitochondrion that resides in the mitochondrial matrix, aconitase (Aco1), tagged with GFP. Again, we observed segregation of the mitochondria in meiotic cells resulting from a cross between cells with unlabeled mitochondria and cells with mitochondria labeled with Aco1-GFP (Fig. S1, C and D).

The segregation of mitochondria that we observed could result from a scenario where mitochondria underwent mixing upon zygote formation but then subsequently demixed via a different process. To test if this occurred, we acquired long-term time-lapse videos of fission yeast cells undergoing meiosis (*n* = 13). Again, we used parental cells with differently labeled mitochondria. We observed that the segregation of mitochondria occurred very early in the meiotic cycle and was maintained during the later stages (Fig. 2 C, segregated; and Video 3, left). In some zygotes, partial mixing of mitochondrial material between the two parents was apparent (Fig. 2 C, partially mixed; and Video 3, right). We quantified the degree of mitochondrial mixing in the early and late stages of meiosis from the data in Fig. 2 B. We observed that the parental mitochondria of half of the zygotes remained segregated, and the other half was partially mixed (Fig. 2 D). None of the zygotes observed displayed complete mixing of mitochondria.

### The anchor protein Mcp5 tethers mitochondria to the poles during prophase I of meiosis

In budding yeast, the Mcp5 homologue Num1 is a part of the MECA structure and is essential for retention of mitochondria in the mother cell, while the Myo2 motor carries mitochondria to the bud on actin cables (Klecker et al., 2013; Lackner et al., 2013). The mitochondrial localization at the poles that we observed (Figs. 1 and 2 A) was reminiscent of the organization of Mcp5 spots at the cortex (Saito et al., 2006; Yamashita and Yamamoto, 2006; Thankachan et al., 2017). Mcp5 clusters into ∼30 foci containing ∼10 molecules per focus, preferentially at the cell poles (Thankachan et al., 2017). Additionally, Mcp5 is a meiosis-specific protein that is expressed predominantly during meiotic prophase in fission yeast, when it anchors dynein to enable oscillations of the horsetail nucleus (Saito et al., 2006; Yamashita and Yamamoto, 2006).

**Figure 3.**
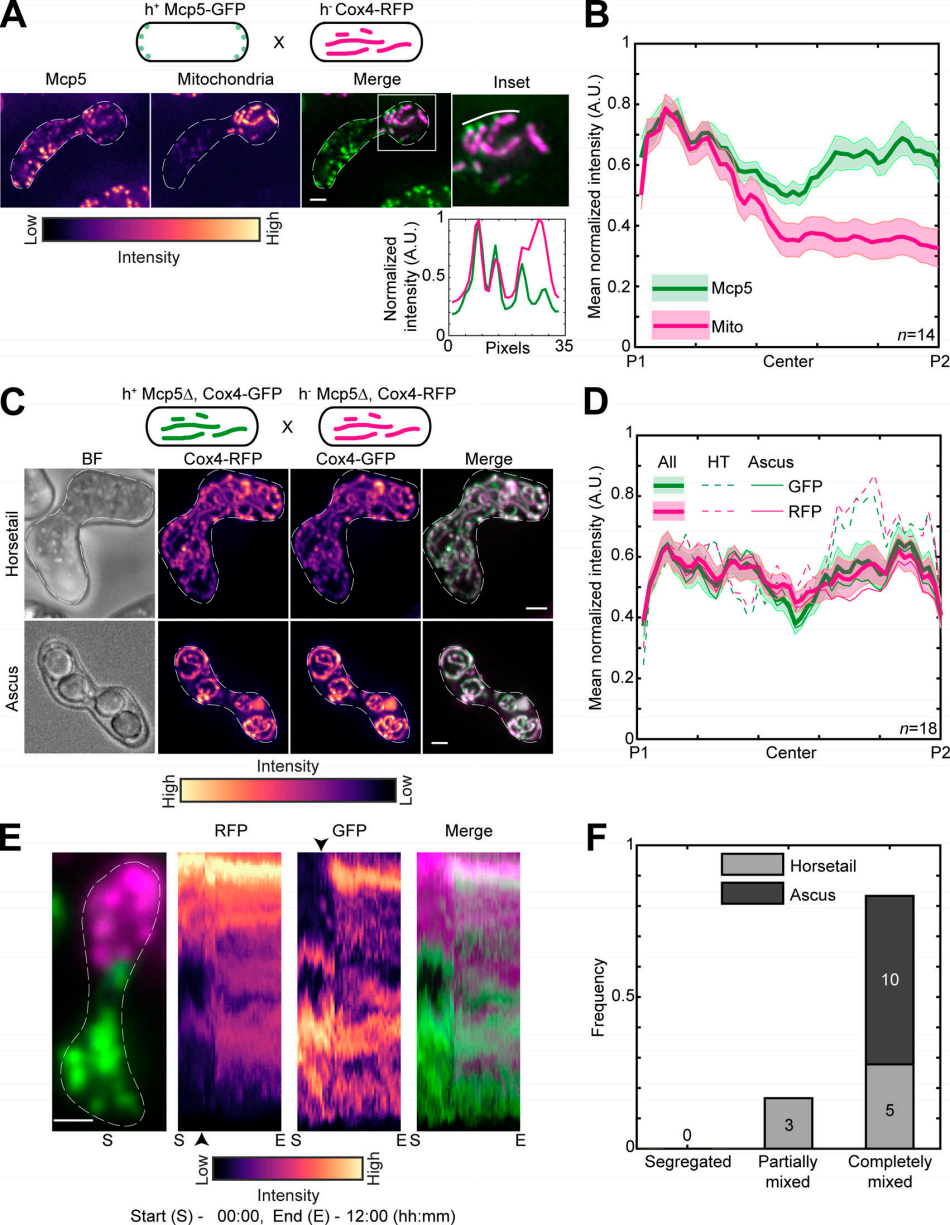
**Mcp5 is essential for mitochondrial tethering to the cortex. (A)** Schematic of the cross performed (top, strain FY16854xPT1651; see Table S1), maximum-intensity–projected images of Mcp5 labeled with GFP (left) and mitochondria labeled with Cox4-RFP (second from left) represented in the intensity map to the bottom of the images, their merge (third from left), and the inset (right). The intensity of mitochondria (magenta) and Mcp5 (green) 2 pixels below the white line marked in the inset appears in the plot below. **(B)** Plot of mean normalized intensities of Mcp5 (green line) and mitochondria (magenta line) across the length of the cell from the cross indicated in A (*n* = 14). **(C)** Schematic of the cross performed (top, strain VA066xVA074; see Table S1), maximum-intensity–projected images of bright-field channel (BF; first from left), mitochondria labeled with Cox4-RFP (second from left) and mitochondria labeled with Cox4-GFP (third from left) represented in the intensity map to the bottom of the images, and their merge (right) during the early stage (horsetail, top) and late stage (ascus, bottom) of meiosis. **(D)** Plot of mean normalized intensities of RFP (magenta lines) and GFP (green lines) in the horsetail stage (HT; dashed lines, *n=*8), ascus stage (Ascus; solid lines, *n =* 10), and the stages combined (All; thick solid lines) across the length of the cell from the cross indicated in C (*n* = 18). **(E)** Representative maximum-intensity–projected image (left) and kymographs of time-lapse movies of RFP channel (second from left), GFP channel (third from left), and their merge (right) of meiotic cells resulting from the cross indicated in C, exhibiting the completely mixed phenotype. The intensity map of kymographs of the GFP and RFP channel is indicated to the bottom of the images. S denotes start of imaging at 00:00, and E denotes end of imaging at 12:00 (hours:minutes). The black arrowheads point to the time when mitochondria start to mix. **(F)** Stacked bar plot of frequency of segregated, partially mixed, and completely mixed phenotypes observed in horsetail (light gray) and ascus (dark gray) stages from the data in D. In A, C, and E, scale bars represent 2 µm and dashed lines represent cell outlines. In B and D, shaded regions represent SEM.

Therefore, to test if mitochondria were also being anchored by Mcp5 in fission yeast, we first visualized zygotes which expressed fluorescently labeled mitochondria and Mcp5. Similar to previous observations, we counted 29.8 ± 11.6 Mcp5 spots per zygote (Thankachan et al., 2017), of which 87.9 ± 7.6% (*n =* 536 Mcp5 spots from 18 cells; Fig. S2 A) colocalized with mitochondria (Kraft and Lackner, 2019). We observed complete colocalization between mitochondria at the cortex and Mcp5 foci (Fig. 3 A). In this cross, GFP-labeled Mcp5 was expressed from only one of the parents, and RFP-labeled Cox4 was expressed from the other. Interestingly, while Mcp5’s signal was visible at both poles of the cell, mitochondrial signal was again restricted to one pole (Fig. 3 B and Video 4), indicating that there were no barriers to diffusion or mixing of other proteins in the zygote. Additionally, mitochondria continued to remain dissociated from the microtubules when bound to Mcp5 (Fig. S2 B), as observed in Fig. 1 A. To verify that the attachment to microtubules was not necessary for segregation during meiosis, we employed parental cells lacking the microtubule-mitochondrial linker protein Mmb1 (Fu et al., 2011). Additionally, one of the parental cells had its mitochondria fluorescently labeled. In zygotes and asci resulting from this cross, we observed that parental mitochondria continued to remain segregated (Fig. S2, C and D).

We then proceeded to set up a cross between cells lacking Mcp5 but with GFP- and RFP-labeled mitochondria. In stark contrast to wild-type zygotes, these Mcp5Δ meiotic cells showed complete mixing of parental mitochondria in both early and late stages (Fig. 3 C and Video 5). These observations were also substantiated by measurement of GFP and RFP intensities across the length of the cell during all stages of meiosis (Fig. 3 D).

We then visualized the dynamics of mitochondrial mixing in these cells lacking Mcp5 using long-term time-lapse imaging and observed that most cells exhibited complete mixing of parental mitochondria (*n =* 13; Fig. 3 E and Video 6). Analysis of the degree of mixing revealed that none of the zygotes displayed segregated mitochondria (Fig. 3 F), contrary to the results obtained in cells containing Mcp5. Further, the expression of Mcp5 from only one of the parents was not sufficient to reverse the mitochondrial mixing phenotype (Fig. S3, A–C). In meiotic cells resulting from a cross between a parental cell containing Mcp5 and the other lacking Mcp5, while some of the early stage cells displayed the segregated phenotype, all of the later stage cells contained a complete mix of parental mitochondria (Fig. S3 D). This likely indicates that the presence of a single copy of Mcp5 in the zygote might be sufficient to delay, but not abolish, mitochondrial mixing.

### Mcp5 uses its coiled-coil (CC) domain to anchor mitochondria to the cortex

**Figure 4.**
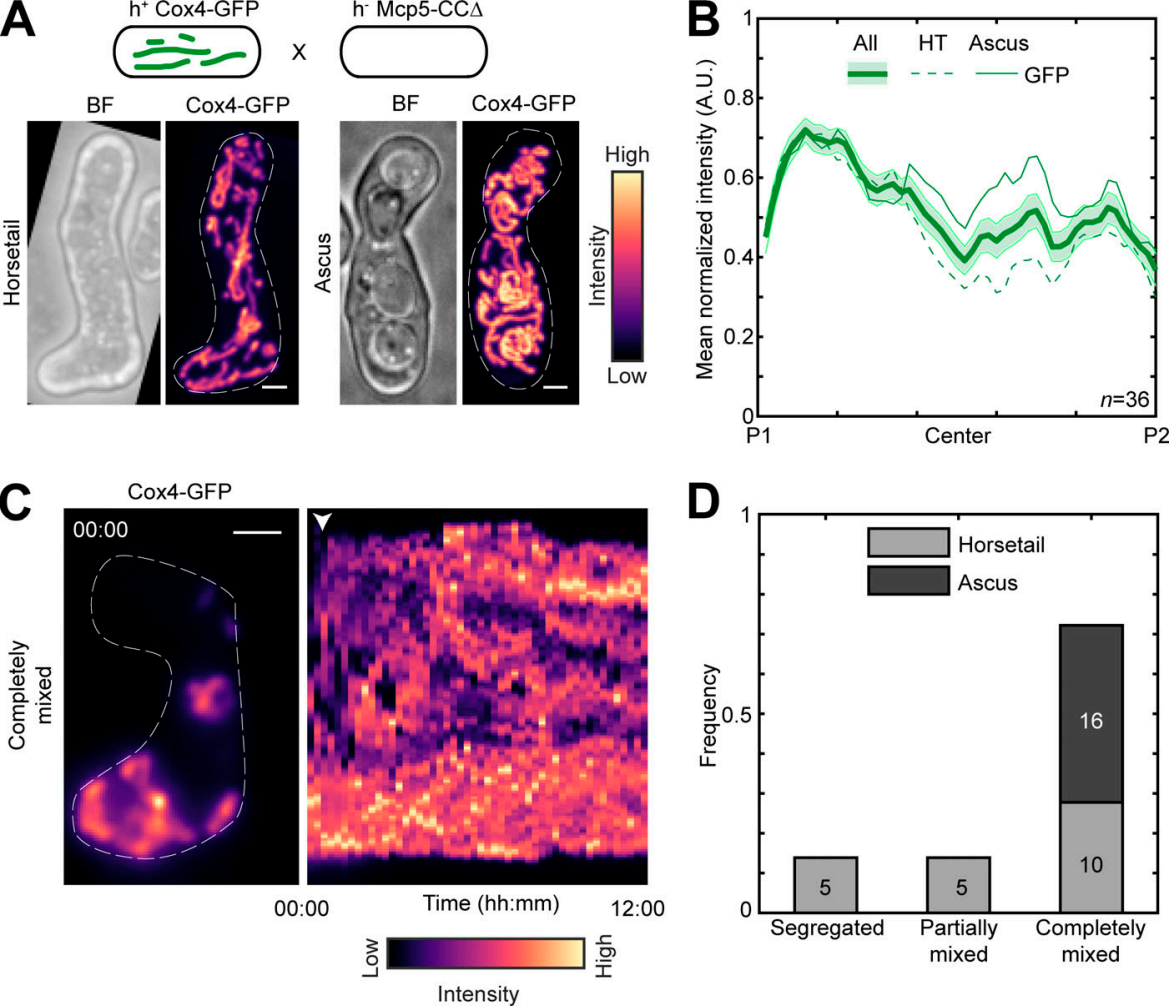
**Mcp5 associates with mitochondria via the CC domain. (A)** Schematic of the cross performed (top, strain PT1650xFY16897; see Table S1), maximum-intensity–projected images of bright-field channel (BF; top) and mitochondria labeled with Cox4-GFP (bottom) during the early stage (horsetail, left) and late stage (ascus, right) of meiosis represented in the intensity map to the right of the images. **(B)** Plot of mean normalized intensity of GFP (green lines) in the horsetail stage (HT; dashed lines, *n =* 20), ascus stage (Ascus; solid lines, *n =* 16), and the stages combined (All; thick solid line) across the length of the cell from the cross indicated in A (*n* = 36). Shaded regions represent SEM. **(C)** Representative maximum-intensity–projected image (left) and kymograph of a time-lapse movie of GFP channel (right) of meiotic cells resulting from the cross indicated in A, exhibiting the completely mixed phenotype. The intensity map is indicated to the bottom of the images. The numbers indicate the timestamp (hours:minutes). The white arrowhead points to the time when mitochondria start to mix. **(D)** Stacked bar plot of frequency of segregated, partially mixed, and completely mixed phenotypes observed in horsetail (light gray) and ascus (dark gray) stages from the data in B. In A and C, scale bars represent 2 µm and dashed lines represent cell outlines.

Mcp5 comprises a pleckstrin-homology domain, which is essential for its attachment to the membrane, and a CC domain, which is required for its binding to dynein (Saito et al., 2006; Yamashita and Yamamoto, 2006; Ananthanarayanan, 2016). We asked if the CC domain was also responsible for Mcp5′s attachment to the mitochondria. To answer this, we visualized mitochondrial distribution in a cross between a parental cell lacking the Mcp5’s CC domain and the other parent containing Mcp5 and fluorescently labeled mitochondria (Fig. 4 A). If mitochondrial tethering by Mcp5-CCΔ was intact, we would observe an intensity pattern similar to that in Fig. S1 A or Fig. S2 A. However, we saw that the fluorescence from the mitochondria was distributed throughout the cell in both early and late stages (Fig. 4 B and Video 7), indicating that Mcp5 indeed uses its CC domain to tether mitochondria to the cortex during meiotic prophase I. Meiotic cells expressing Mcp5-CCΔ additionally displayed mixing of mitochondria (Fig. 4, C and D; and Video 8) similar to that seen in Mcp5Δ cells (Fig. S3, C and D).

### Dynein-Mcp5 spots on the membrane are devoid of mitochondria

**Figure 5.**
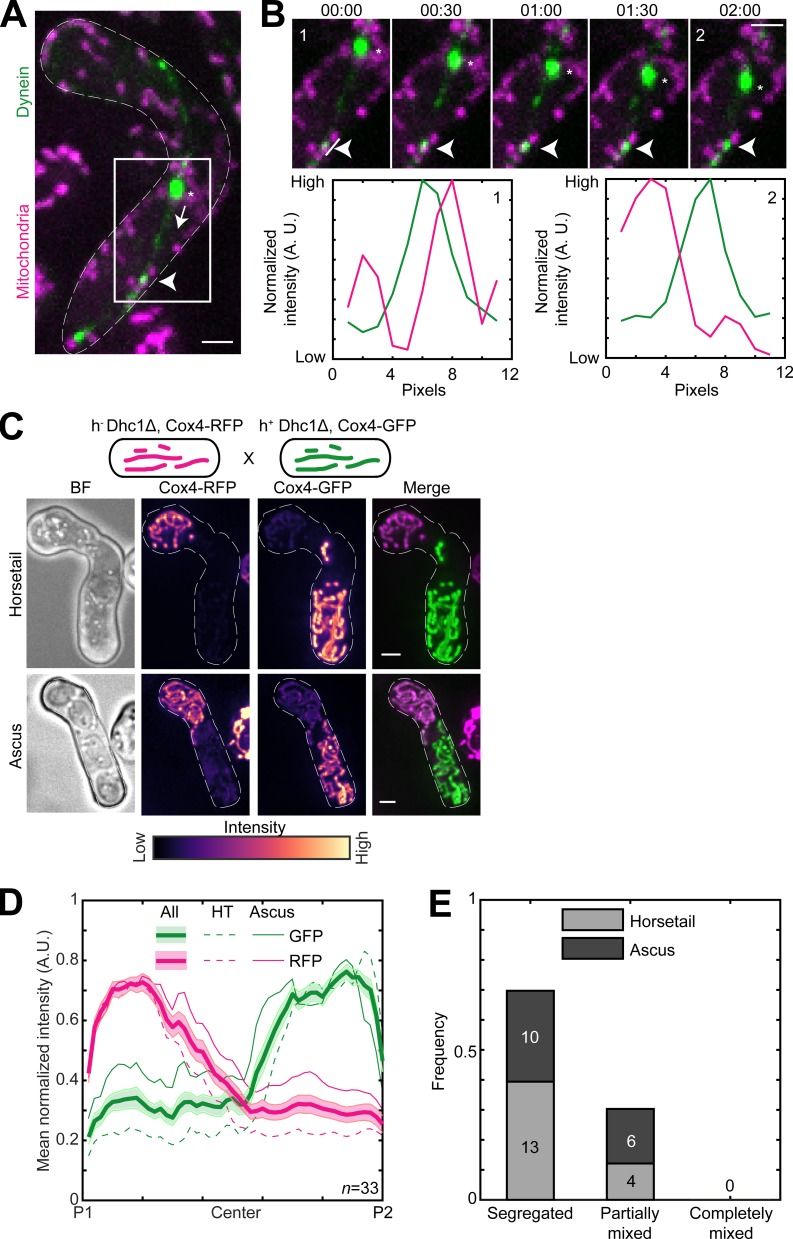
**Dynein and mitochondria do not bind to the same Mcp5 foci. (A)** Maximum-intensity–projected images showing a merge of dynein (green) and mitochondria (magenta) in a meiotic cell undergoing nuclear oscillations (left, strain VA099; see Table S1). The white arrowhead points to a representative dynein spot on the cortex, the asterisk indicates the position of the SPB, and the dashed arrow points to the direction of SPB movement. **(B)** Montage of the inset indicated in A with dynein in green and mitochondria in magenta (top) and plots of normalized intensity (bottom) of dynein (green) and mitochondria (magenta) 1 pixel to the left of the line indicated in montage numbered 1 (left) and numbered 2 (right). The white arrowheads point to the dynein spot. Time is indicated above the images of the montage in minutes:seconds. **(C)** Schematic of the cross performed (top, strain VA091xVA092; see Table S1), maximum-intensity–projected images of the bright-field channel (BF; first from left), mitochondria labeled with Cox4-RFP (second from left) and mitochondria labeled with Cox4-GFP (third from left) represented in the intensity map to the bottom of the images, and their merge (right) during the early stage (horsetail, top) and late stage (ascus, bottom) of meiosis. **(D)** Plot of mean normalized intensities of RFP (magenta lines) and GFP (green lines) in the horsetail stage (HT; dashed lines, *n =* 17), ascus stage (Ascus; solid lines, *n =* 16), and the stages combined (All; thick solid lines) across the length of the cell from the cross indicated in C (*n* = 33). Shaded regions represent SEM. **(E)** Stacked bar plot of frequency of segregated, partially mixed and completely mixed phenotypes observed in horsetail (light gray) and ascus (dark gray) stages from the data in D. In A–C, scale bars represent 2 µm and dashed lines represent cell outlines.

Mcp5 was originally identified as a cytoplasmic dynein anchor during meiotic nuclear oscillations in fission yeast (Saito et al., 2006; Yamashita and Yamamoto, 2006). Here, we have identified an additional role for Mcp5 in anchoring mitochondria. In both instances, Mcp5 employs its CC domain to serve as a membrane anchor. It is therefore unclear if an Mcp5 spot is capable of simultaneously anchoring both dynein and mitochondria. Therefore, we acquired time-lapse images of zygotes expressing fluorescently labeled dynein and mitochondria that were in the horsetail oscillations phase (Fig. 5 A and Video 9). We observed that 87% of anchored dynein spots (*n* = 29 dynein spots from 23 cells) that were involved in the movement of the spindle pole body (SPB) and the attached nucleus did not colocalize with mitochondria (Fig. 5 B), indicating that Mcp5 foci that anchored dynein were typically precluded from tethering mitochondria.

Additionally, when deleting Mcp5 to test its role in mitochondrial tethering, we not only knocked down Mcp5 but also abrogated the oscillations that occur during the meiotic prophase (Saito et al., 2006; Yamashita and Yamamoto, 2006; Thankachan et al., 2017). To delineate the specific role of the oscillations, if any, in facilitating parental mitochondrial segregation, we sought to attenuate the oscillations of the horsetail nucleus while keeping Mcp5 intact. To this end, we employed cells lacking the motor protein dynein, which is essential to power the oscillations (Yamamoto et al., 1999) but has no effect on Mcp5 localization at the cortex (Saito et al., 2006; Yamashita and Yamamoto, 2006). We set up a cross between parental cells containing a deletion of the dynein heavy chain (Dhc1) gene but containing differently labeled mitochondria and visualized the distribution of mitochondria in the resulting zygotes and asci (Fig. 5 C). We observed that the parental mitochondria remained predominantly segregated in both horsetail zygotes as well and asci (Figs. 5, D and E; and Video 10), indicating that the nuclear oscillations had no role to play in the segregation of parental mitochondria. Further, the absence of dynein in these cells might explain the slightly better mitochondrial segregation phenotype that we observed in Fig. 5 (C–E), since the lack of dynein in these zygotes made a few more Mcp5 foci available for binding by the mitochondria.

### MtDNA is uniparentally inherited

**Figure 6.**
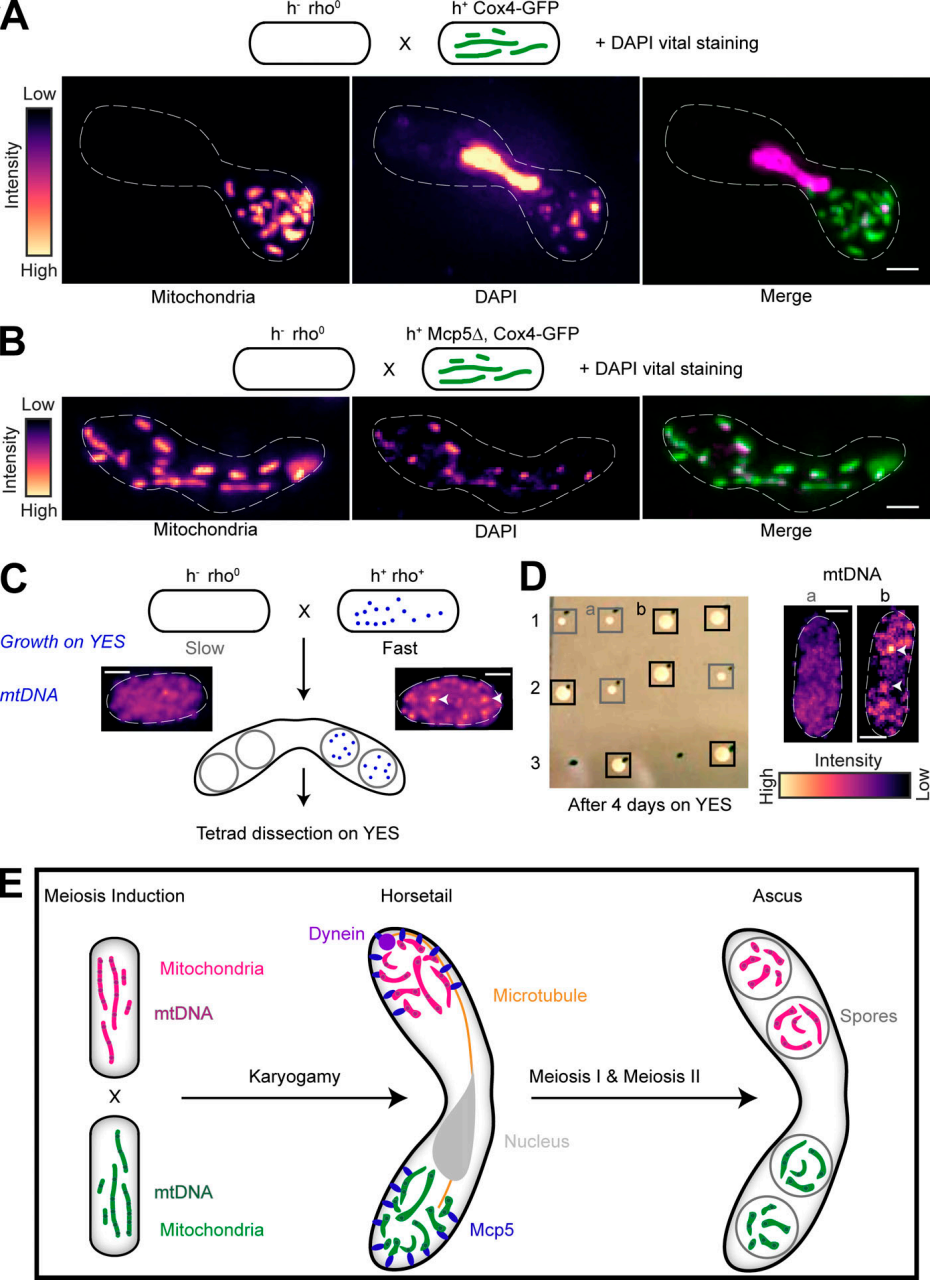
**mtDNA are uniparentally inherited during fission yeast meiosis. (A)** Schematic of the cross and DAPI vital staining performed (top, strain PHP14xPT1650; see Table S1), maximum-intensity–projected images of mitochondria labeled with Cox4-GFP (left) and mtDNA (DAPI, center) represented in the intensity map to the left of the images and their merge (right). Note that a small portion of zygotes exhibit nuclear DAPI signal during vital staining. One such zygote has been chosen here to demonstrate that the mitochondria and mtDNA remain segregated even upon complete fusion of the zygote, as indicated by the horsetail nucleus. **(B)** Schematic of the cross and DAPI vital staining performed (top, strain PHP14xVA074; see Table S1), maximum-intensity–projected images of mitochondria labeled with Cox4-GFP (left) and mtDNA (DAPI, center) represented in the intensity map to the left of the images, and their merge (right). **(C)** Schematic of the rho^0^ and rho^+^ cross performed to obtain asci, followed by tetrad dissection and growth on YES plates. Maximum-intensity–projected images of vital DAPI staining show absence of mtDNA in rho^0^ cells (left) and presence in rho^+^ cells (right, white arrowheads). Note that rho^0^ cells grow slower than rho^+^ cells on rich medium. **(D)** Image of colonies formed on YES (left) following tetrad dissection of asci formed from the cross indicated in A, and maximum-intensity–projected images of vital DAPI staining (right) in representative slow-growing (gray boxes, a) and fast-growing (black boxes, b) colonies, with mtDNA indicated with white arrowheads. **(E)** Schematic of uniparental mitochondrial inheritance in fission yeast mediated by the tethering of parental mitochondria to the cortex by the anchor protein Mcp5. In A–D, scale bars represent 2 µm and dashed lines represent cell outlines.

To confirm that the segregation of parental mitochondria resulted in segregation of the parental mtDNA, we first set up a cross between a cell expressing fluorescently labeled mitochondria and a cell lacking mtDNA nucleoids (rho^0^; Haffter and Fox, 1992). Then, we labeled mtDNA by vital DAPI staining of the resulting zygote (Williamson and Fennell, 1979). In such a scenario, all the mtDNA in the products of this cross would originate from the non-rho^0^ (rho^+^) parental cell. Accordingly, we again observed mitochondrial segregation in the zygote and also observed complete colocalization between the labeled mitochondria and mtDNA (*n* = 11; Fig. 6 A). In contrast, in a cross between Mcp5Δ cells with fluorescently labeled mitochondria and rho^0^ cells, we observed localization of mitochondria and mtDNA throughout the zygote (*n* = 13; Fig. 6 B). However, we observed that asci were refractory to the vital DAPI stain and therefore employed tetrad dissection to understand mtDNA inheritance pattern in the progeny of meiosis in the presence and absence of Mcp5.

Cells lacking mtDNA nucleoids grow much slower on rich media than rho^+^ cells (Fig. 6 C; Haffter and Fox, 1992). We employed this difference in growth rate between rho^0^ and rho^+^ cells to understand the segregation of mtDNA during meiosis in *S. pombe.* If mtDNA were segregated in a pattern similar to that observed of mitochondria (Fig. 2), then a cross between rho^0^ and rho^+^ cells would result in two of the spores containing mtDNA and the other two lacking mtDNA (Fig. 6 C). When these spores are isolated following formation of spores and tetrad dissection, we would expect to observe normal growth of the two spores that inherited the mtDNA from the parental rho^+^ cell and slower growth of the two spores that did not inherit mtDNA (Fig. 6 C). Accordingly, we observed that 72.2% of the dissected tetrads (*n =* 18 tetrads) resulting from a cross between rho^0^ and rho^+^ cells containing Mcp5 (strain PHP4xPT1650; see Table S1) exhibited a phenotype of mtDNA segregation to two spores alone. Of the four spores from these tetrads, two spores grew faster on rich medium (yeast extract plus supplements [YES] agar plate; see Materials and methods) than the other two (Fig. 6 D, rows 1 and 2), and in some instances two of the four spores failed to grow at all 4 d after growth on YES medium (Fig. 6 D, row 3). Additionally, visualization of mtDNA in the former revealed that fast-growing cells exhibited mtDNA (92.3%, *n* = 13 spores), whereas slow-growing cells lacked mtDNA (83.3%, *n* = 6 spores; Fig. 6 D, right), indicating that the presence or absence of mtDNA could be reliably linked to fast and slow growth of spores, respectively. These results confirmed that mtDNA segregated predominantly to only two of the four spores.

In the absence of Mcp5 in one of the parents of meiosis, mitochondria appeared completely mixed in the ascus stage (Fig. S3). Accordingly, in the absence of Mcp5 in rho^+^ parental strain (strain PHP4xVA074; see Table S1), only 31.3% of the tetrads dissected (*n =* 16 tetrads) exhibited mtDNA segregation similar to that observed in Fig. 6 D. Again, 91.7% of fast-growing cells (*n* = 12 spores) exhibited mtDNA, and 75% (*n* = 4 spores) of slow-growing cells lacked mtDNA. Taken together, we observed that Mcp5 was essential for the preferential inheritance of mitochondria and mtDNA from one of the parental strains. A schematic summarizing these results is depicted in Fig. 6 E.

## Discussion

Uniparental mitochondrial inheritance is a common feature among several eukaryotes, including unicellular fungi such as *Crytptococcus neoformans* and *Ustilago maydis*. In *C. neoformans*, mitochondria from the MATa parent are selectively passed on to progeny by an as-yet-unknown degradation mechanism that affects the MATα mitochondria (Yan and Xu, 2003; Yan et al., 2007). In *U. maydis,* the a2 strain, and not a1, contributes all of the mitochondria by using a mechanism that protects a2 mitochondria from degradation due to the interaction of two genes at the a2 mating type locus, Rga2 and Lga2 (Fedler et al., 2009). In mammalian cells, sperm mitochondria typically enter the oocyte post fertilization, but then undergo selective ubiquitination and proteolysis thereby effecting maternal mitochondrial inheritance in the progeny (Sutovsky et al., 1999, 2000).

Here, we have discovered that the unicellular yeast, *S. pombe* also undergoes uniparental mitochondrial inheritance. The progeny of a meiotic cross are thus homoplasmic for either the h^+^ or h^−^ parental mitochondria and mtDNA. *S. pombe* achieves uniparental inheritance by using the anchor protein Mcp5 to tether mitochondria to the cortex during meiotic prophase. While this mechanism relies on segregating mitochondria by their anchoring to the cortex, other segregation methods are also possible such as the chloroplast inheritance mechanism in the green alga *Cylindrocystis*, where the two chloroplasts from each parent in the zygote do not mix or divide and are then individually distributed to the four meiotic products (Smith, 1950).

In *S. cerevisiae*, Num1 and Mdm36, which are key components of MECA, serve to anchor mitochondria in the mother cell during mitotic anaphase (Lackner et al., 2013). Num1 also tethers mitochondria to the cortex during the early stages of *S. cerevisiae* meiosis, but mitochondria dissociate from the cortex in meiosis II due to the programmed destruction of MECA by Ime2-dependent phosphorylation (Sawyer et al., 2019). In *S. pombe*, the expression profile of Mcp5 peaks during meiotic prophase (Mata et al., 2002; Saito et al., 2006; Yamashita and Yamamoto, 2006) ensuring that mitochondria are anchored to the cortex during the earliest stages of meiosis.

In budding yeast, Num1 cluster formation requires mitochondrial attachment and the resulting clusters of Num1 are required for dynein anchoring (Lammers and Markus, 2015; Kraft and Lackner, 2017; Schmit et al., 2018). A recent study has also established the role of *S. pombe* Mcp5 and its CC domain in tethering mitochondria during fission yeast meiosis (Kraft and Lackner, 2019). However, contrary to our observations, an individual Mcp5 spot was found to be able to tether mitochondria and dynein simultaneously. This discrepancy likely arises from the difference in analysis procedures followed to ascertain colocalization. In this work, we used 3D reconstructed images coupled with intensity profile mapping (see Materials and methods) to rule out artifacts due to analysis in single focal planes.

Additionally, *S. cerevisiae* Num1 clusters might accommodate both mitochondria and dynein by making a fraction of molecules in the clusters available for dynein binding after mitochondrial association. In fission yeast, the number of dynein molecules that form a cluster is approximately equal to the number of Mcp5 molecules that make up a focus at the cortex (Ananthanarayanan et al., 2013; Thankachan et al., 2017). Therefore, our results are likely a reflection of the stoichiometry of binding between Mcp5 and dynein that does not allow for mitochondrial binding to a preexisting Mcp5-dynein spot.

In conclusion, we report that fission yeast achieves uniparental mitochondrial inheritance by anchoring and thereby segregating parental mitochondria during the earliest stages of meiosis. Future studies will help us understand what the role of uniparental inheritance is in wild-type cells and what the consequence of perturbation of this phenomenon would be, particularly in context of deleterious mtDNA mutations.

## Materials and methods

### Strains and media

The fission yeast strains used in the study are listed in Table S1. Fission yeast cells were grown on yeast extract medium or Edinburgh minimal medium (EMM) with appropriate supplements (Forsburg and Rhind, 2006).

### Construction of strains

Strain VA019 was constructed by crossing strain MTY271 (*h^−^ mCherry-atb2:hphMX6 leu1-32 ura-d18*; see Table S1) with strain FY16887 (*h^90^ leu1-32 (mcp5::ura4+)::GFP-mcp5*; see Table S1) following the random spore analysis protocol (Forsburg and Rhind, 2006). Similarly, strain VA066 was constructed by crossing strain PT1651 (*h*^*−*^ *cox4-RFP:leu1 ade6-M210 leu1-32 ura4-D18*; see Table S1) with strain FY16839 (*h^90^ leu1-32 ura4-D18 mcp5::ura4+*; see Table S1), strain VA074 was constructed by crossing strain PT1650 (*h^+^ cox4-GFP:leu1 ade6-M210 leu1-32 ura4-D18*; see Table S1) with strain FY16839 (*h^90^ leu1-32 ura4-D18 mcp5::ura4+*; see Table S1), strain VA080 was constructed by crossing strain PT2244 (*h^+^ mmb1Δ:Kanr cox4-GFP:leu2 mCherry-atb2:Hygr ade6-m210 leu1-32 ura4-d18*; see Table S1) with strain L972 (*h^−^ WT*; see Table S1), strain VA086 was constructed by crossing strain PT1651 (*h^−^ cox4-RFP:leu1 ade6-M210 leu1-32 ura4-D18*; see Table S1) with strain FY6871 (*h^+^ ade6-M210 ura4-D18 leu1*; see Table S1), strain VA091 was constructed by crossing strain PT1650 (*h^+^ cox4-GFP:leu1 ade6-M210 leu1-32 ura4-D18*; see Table S1) with strain FY21150 (*h^−^ leu1 ura4 dhc1Δ::ura4 (DHC106-1)*; see Table S1), strain VA092 was constructed by crossing strain VA086 (*h^+^ cox4-RFP:leu1 ade6-M210 ura4-D18*; see Table S1) with strain FY21150 (*h*^−^* leu1 ura4 dhc1Δ::ura4 (DHC106-1)*; see Table S1), and strain VA099 was constructed by crossing strain SV56 (*h^90^ dhc1-3xGFP:kan r leu1-32 lys1 ura4-D18*; see Table S1) with strain PT1651 (*h^−^ cox4-RFP:leu1 ade6-M210 leu1-32 ura4-D18*; see Table S1).

### Induction of meiosis and preparation of cells for imaging

Meiosis was induced in h^90^ strains by suspending a loopful of cells in 100 µl of 0.85% NaCl and spotting on to sporulation agar plates. For a cross between h^+^ and h^−^, equal amounts of parental strains were resuspended in NaCl and spotted onto a sporulation agar plate. The plate was incubated for ∼8 h and ∼15 h at room temperature for h^90^ and h^+^/h^−^ cross, respectively, before imaging. For imaging, cells were resuspended in EMM-N and aspirated onto a 2 mg/ml lectin (catalog no. L2380; Sigma-Aldrich)–coated 0.17-mm glass-bottom dish (catalog no. 100350; SPL). Cells were allowed to adhere to the glass bottom for 15–20 min. Unattached cells were washed out and cells were imaged in EMM-N.

### MitoTracker staining

For staining mitochondria in Fig. S2 A, meiotic cells were washed once with autoclaved water, and stained with 200 nM MitoTracker Orange CMTMRos (catalog no. M7510; Thermo Fisher Scientific) dissolved in EMM-N for 20 min. After this, cells were washed thrice with EMM before imaging. Mitochondiral staining was carried out similarly in Fig. S2 B with MitoTracker Deep Red FM (catalog no. M22426; Thermo Fisher Scientific).

### DAPI vital staining

Staining of mtDNA in live cells was performed using DAPI as described previously (Williamson and Fennell, 1979). Briefly, cells were washed once with water, resuspended in EMM-N containing 10 µg/ml DAPI (catalog no. D9542; Sigma-Aldrich), and allowed to incubate at 30°C for 45 min, with shaking at 200 rpm. The cells were then washed again with water before proceeding with imaging.

### Microscopy

All images except those in Figs. 4 A, 6 D, and S3 A were obtained and deconvolved using a Deltavision RT microscope (Applied Precision) with a 100×, oil-immersion 1.4 NA objective (Olympus). Excitation of fluorophores was achieved using InsightSSI (Applied Precision) and corresponding filter selection for excitation and emission of DAPI, GFP, RFP, and MitoTracker Deep Red. Z-stacks with 0.2-µm step sizes encompassing the entire cell were captured using a CoolSnapHQ camera (Photometrics) with 2 × 2 binning. The system was controlled using softWoRx 3.5.1 software (Applied Precision) and the deconvolved images were obtained using the built-in setting for each channel. The time-lapse images in Figs. 2 C, 3 E, 4 C, and S3 C were obtained using the confocal mode in the InCell Analyzer-6000 (GE Healthcare) with 60×/0.7 NA objective fitted with an sCMOS 5.5MP camera having an x-y pixel separation of 108 nm. For GFP and RFP imaging, 488- and 561-nm laser lines and bandpass emission filters 525/20 nm and 605/52 nm, respectively, were used. The cells chosen for these time lapses were just about to fuse or already in the horsetail stage and were imaged until sporulation or beyond, for a total of 12 h. These time lapses were captured with a time interval of 15 min and were corrected for bleaching upon acquisition using the histogram-matching algorithm of Fiji.

The images in Figs. 4 A, 6 D, and S3 A were obtained using an inverted microscope (Eclipse Ti2-E; Nikon) fitted with a spinning disk (CSU-X1; Yokogawa), equipped with an EMCCD camera (iXon Ultra-897; Andor) using 488- and 561-nm laser illumination (Toptica) and bandpass filters of 525/35 nm and 617/73 nm, respectively, for GFP and RFP emission, with a 100× oil-immersion 1.49 NA objective (Nikon). Z-stacks were obtained with a step size of 0.2 µm to encompass the entire cell. The time-lapse images in Fig. 5 A were obtained using the spinning disk confocal microscope with a time interval of 30 s between consecutive Z-stacks (with 0.5 µm step size).

### Intensity profile measurement

The intensity of mitochondria and Mcp5 along the length of cells was obtained in Fiji/ImageJ by measuring the average intensity across a segmented line 25–30 pixels in width drawn along the center of the long axis of the cell in maximum-intensity projected images. The intensity profile plots were then generated after normalizing the intensity values to the maximum intensity of that cell. For analysis of colocalization of mitochondria or dynein with Mcp5, the average intensity of mitochondria and Mcp5 along a 3-pixel-wide line centered on an Mcp5 spot was considered in 3D reconstructed images (Fiji’s 3D-project function). The intensities were again normalized to the maximum intensity within each channel in a cell. If the peaks of Mcp5 and mitochondria or dynein were within a pixel of each other, the signals were considered to colocalize. Otherwise, the signals were considered to not colocalize.

### Tetrad dissection

Tetrad dissection was performed with a dissection microscope (SporePlay; Singer Instruments) using a standard protocol as described previously (Ekwall and Thon, 2017). The dissected spores were then allowed to grow on YES agar plates (Forsburg and Rhind, 2006) for at least 4 d before examining them for mtDNA segregation phenotype. Note that spores were not always dissected in the order in which they appeared within the ascus.

### Image analysis and plotting

Intensity profiles were obtained using Fiji/ImageJ software (Schindelin et al., 2012; Rueden et al., 2017). Analysis was performed using custom functions written in MATLAB (MathWorks). All plots were created using MATLAB.

### Online supplemental material

Fig. S1 shows that parental mitochondria remain segregated during meiosis. Fig. S2 shows that mitochondria associate with Mcp5, but not microtubules, during meiosis. Fig. S3 shows that Mcp5 is essential for mitochondrial anchoring during meiosis. Table S1 lists yeast strains used in this study. Video 1 shows a 3D projection of microtubules and mitochondria in a cross between strain KI001 and PT1651 and a 3D projection of the nucleus and mitochondria in a cross of strain FY15112. Video 2 shows 3D projections of GFP-labeled mitochondria and RFP-labeled mitochondria in a cross between strains PT1650 and PT1651. Video 3 shows live-cell confocal microscopy of a cross between strains PT1650 and PT1651. Video 4 shows a 3D projection of Mcp5 and mitochondria in a cross between strains FY16854 and PT1651. Video 5 shows 3D projections of GFP-labeled mitochondria and RFP-labeled mitochondria in a cross between strains VA066 and VA074. Video 6 shows live-cell confocal microscopy of a cross between strains VA066 and VA074. Video 7 shows 3D projections of mitochondria in a cross between strains FY16897 and PT1650. Video 8 shows live-cell confocal microscopy of a cross between strains FY16897 and PT1650. Video 9 shows live-cell spinning disk confocal microscopy of zygotes from strain VA099 with fluorescent dynein and mitochondria. Video 10 shows 3D projections of GFP-labeled mitochondria and RFP-labeled mitochondria in a cross between strains VA091 and VA092.

## Supplementary Material

Supplemental Materials (PDF)

Video 1

Video 2

Video 3

Video 4

Video 5

Video 6

Video 7

Video 8

Video 9

Video 10
