# Supplementary material for: Cortical tethering of mitochondria by the anchor protein Mcp5 enables uniparental inheritance
Source: J Cell Biol. 2019 Oct 3;218(11):3560–71. doi: 10.1083/jcb.201901108 (PMC6829665; doi:10.1083/jcb.201901108)
Supplement: Supplemental Materials (PDF) [file JCB_201901108_sm.pdf]

Supplemental material

Chacko et al., https://doi.org/10.1083/jcb.201901108

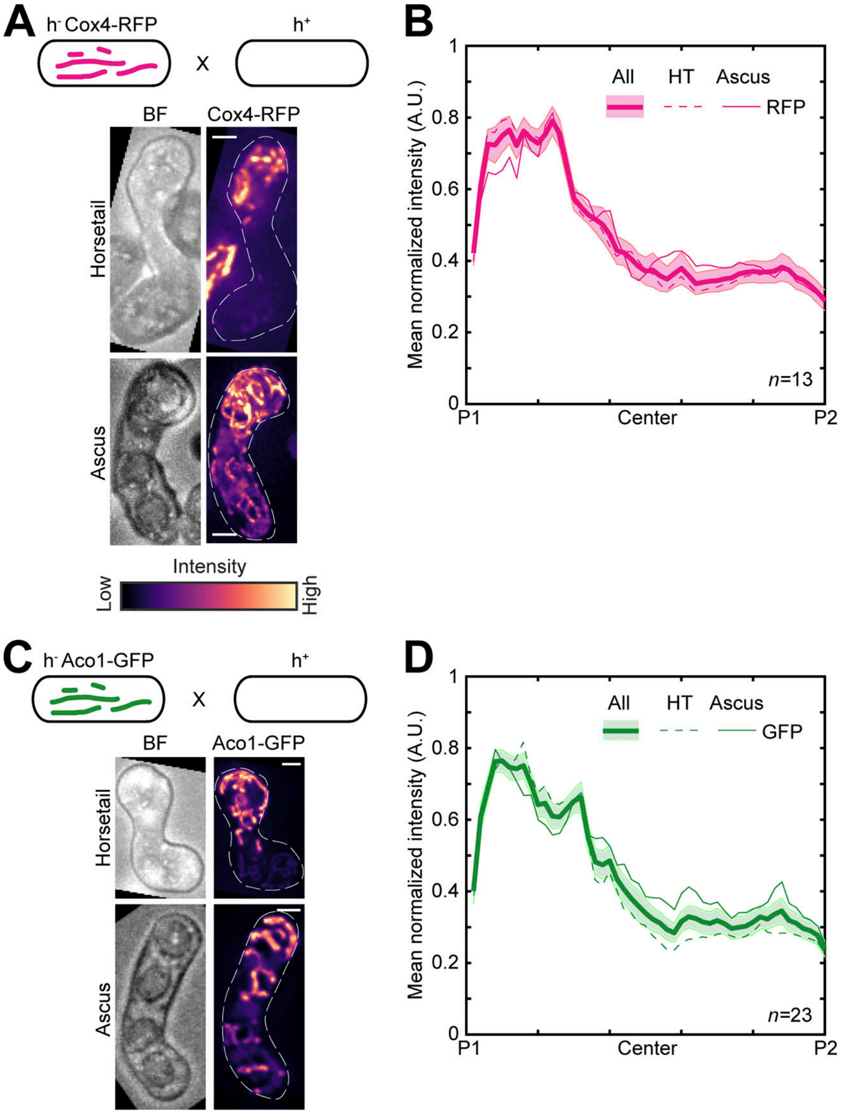

Figure S1. **Parental mitochondria remain segregated during meiosis (related to Fig. 2). (A)** Schematic of the cross performed (top, strain PT1651xL975; see Table S1), maximum-intensity–projected images of bright-field channel (BF; left) and mitochondria labeled with Cox4-RFP (right) during the early stage (horsetail, top) and late stage (ascus, bottom) of meiosis represented in the intensity map to the bottom of the images. **(B)** Plot of mean normalized intensity of RFP (magenta lines) in the horsetail stage (HT; dashed line, n = 9), ascus stage (Ascus; solid line, n=4), and the stages combined (All; thick solid line) across the length of the cell from the cross indicated in A (n = 13). **(C)** Schematic of the cross performed (top, strain MM3264xL975; see Table S1), maximum-intensity–projected images of bright-field channel (BF, left) and mitochondria (right) labeled with Aco1-GFP during the early stage (horsetail, top) and late stage (ascus, bottom) of meiosis represented in the intensity map to the top of the images. **(D)** Plot of mean normalized intensity of GFP (green lines) in the horsetail stage (HT; dashed line, n=13), ascus stage (Ascus; solid line, n = 10), and the stages combined (All; thick solid line) across the length of the cell from the cross indicated in C (n = 23). In A and C, scale bars represent 2 µm and dashed lines represent cell outlines. In B and D, shaded regions represent SEM.

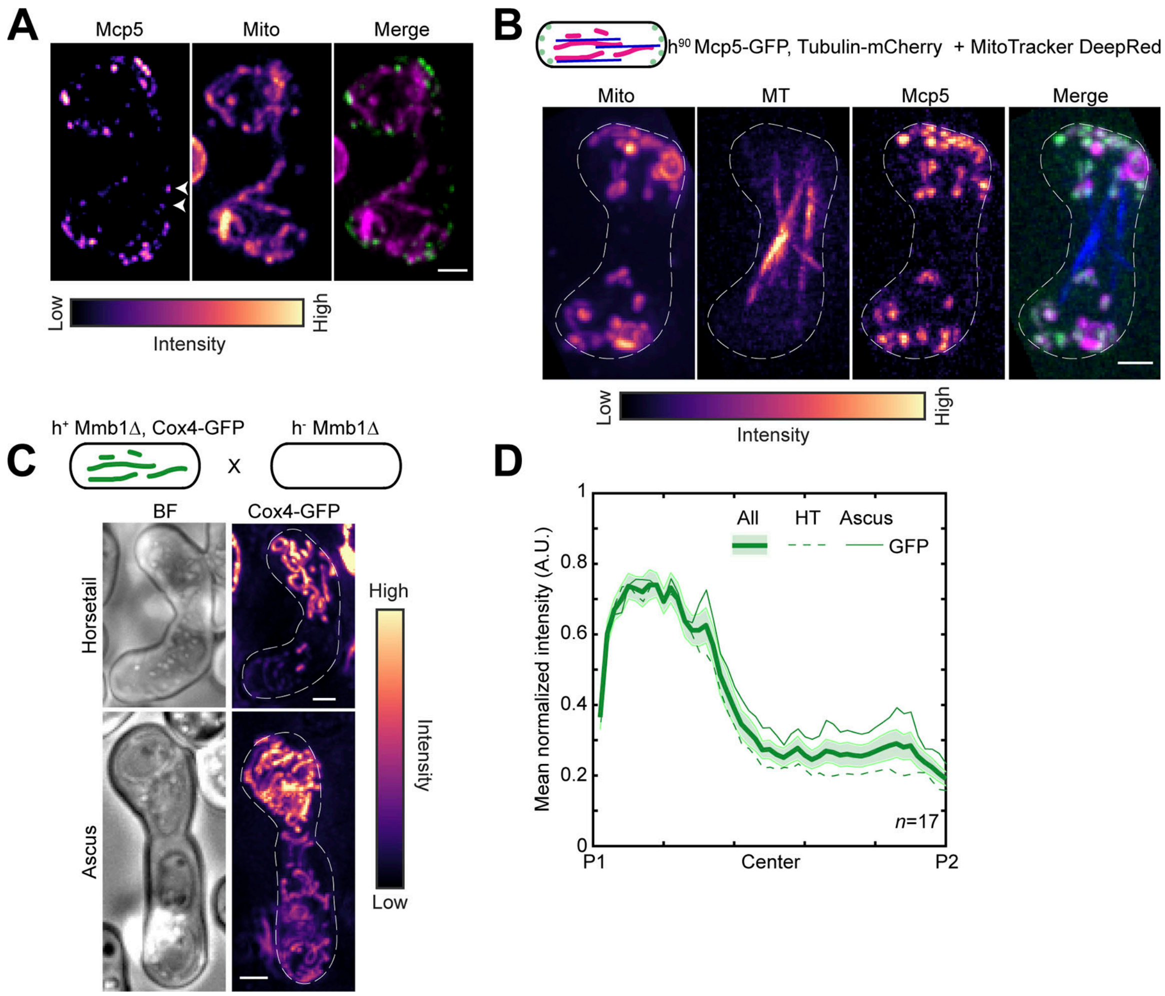

Figure S2. **Mitochondria associate with Mcp5, but not microtubules, during meiosis. (A)** Maximum-intensity–projected images of Mcp5 (left), mitochondria (Mito; center) represented in the intensity map to the bottom of the images, and their merge (right). The white arrowheads point to Mcp5 spots that do not colocalize with mitochondria. Scale bars represent 2 µm. **(B)** Representative maximum-intensity–projected images of mitochondria (Mito; first from left), microtubules (MT; second from left), and Mcp5 (third from left) represented in the intensity map to the right of bottom of the images and their merge (right). A schematic of the fluorescence tags is to the top of the images. **(C)** Schematic of the cross performed (top, strain VA080xPT2244; see Table S1), maximum-intensity–projected images of bright-field channel (BF; left) and mitochondria (right) labeled with Cox4-GFP during the early stage (horsetail; top) and late stage (ascus; bottom) of meiosis represented in the intensity map to the right of the images. **(D)** Plot of mean normalized intensity of GFP (green lines) in the horsetail stage (HT; dashed line, *n* = 9), ascus stage (Ascus; solid line, *n* = 8), and the stages combined (All; thick solid line) across the length of the cell from the cross indicated in C (*n* = 17). The shaded region represents SEM. In B and C, scale bars represent 2 µm and dashed lines represent cell outlines.

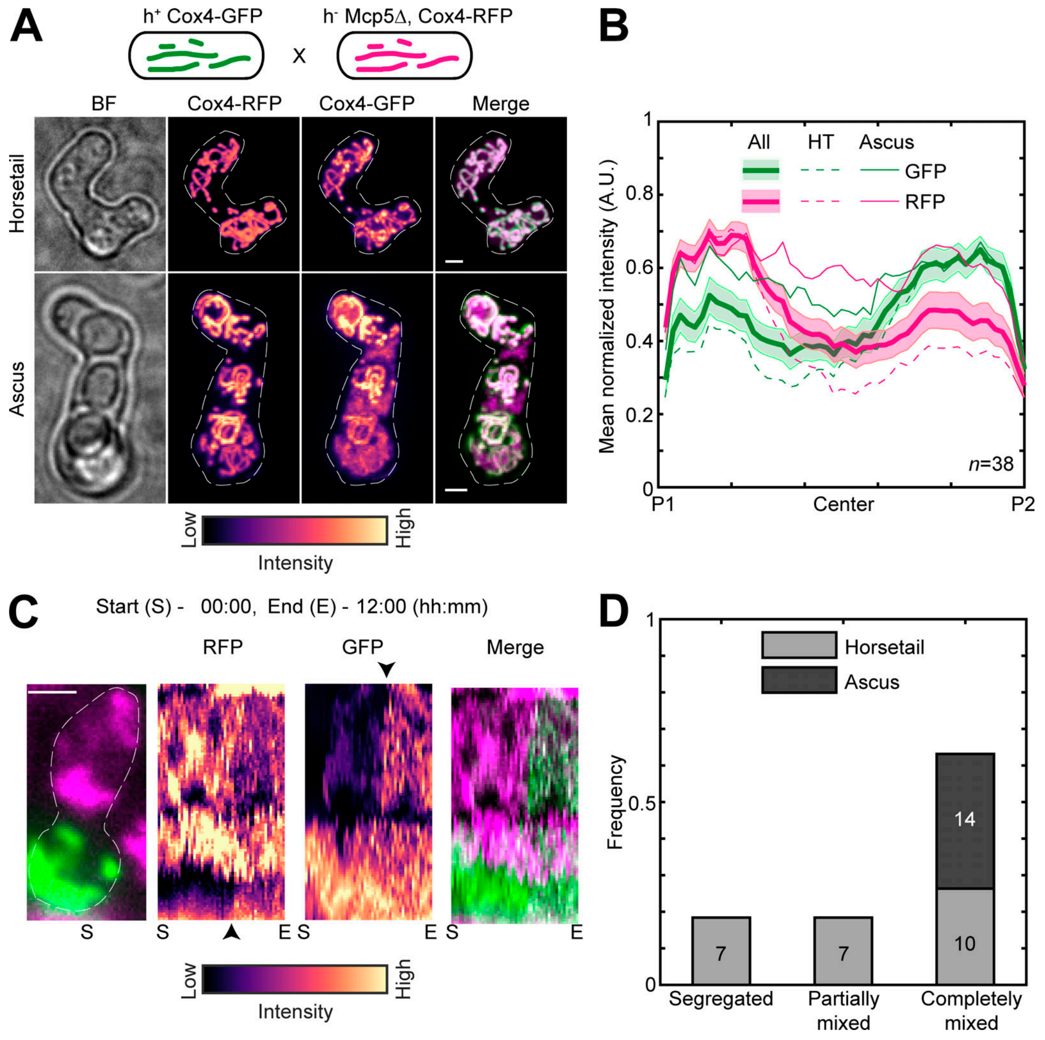

Figure S3. **Mcp5 is essential for mitochondrial anchoring during meiosis. (A)** Schematic of the cross performed (top, strain PT1650xVA066; see Table S1), maximum-intensity–projected images of bright-field channel (BF; first from left), mitochondria labeled with Cox4-RFP (second from left) and mitochondria labeled with Cox4-GFP (third from left) represented in the intensity map to the bottom of the images, and their merge (right) during the early stage (horsetail, top) and late stage (ascus, bottom) of meiosis. **(B)** Plot of mean normalized intensities of RFP (magenta lines) and GFP (green lines) in the horsetail stage (HT; dashed line, *n* = 24), ascus stage (Ascus; solid line, *n* = 14), and the stages combined (All; thick solid lines) across the length of the cell from the cross indicated in A (*n* = 38). The shaded region represents SEM. **(C)** Representative maximum-intensity–projected image (left) and kymographs of a time-lapse movie of RFP channel (second from left), GFP channel (third from left), and their merge (right) of meiotic cells resulting from the cross indicated in A, exhibiting the completely mixed phenotype. The intensity map of kymographs of the GFP and RFP channel is indicated to the bottom of the images. S denotes start of imaging at 00:00, and E denotes end of imaging at 12:00 (hours:minutes). The black arrowheads point to the time when mitochondria start to mix. **(D)** Stacked bar plot of frequency of segregated, partially mixed, and completely mixed phenotypes observed in horsetail (light gray) and ascus (dark gray) stages from the data in B. In A and C, scale bars represent 2 µm and dashed lines represent cell outlines.

Table S1. **Yeast strains used in this study**

Name	Genotype	Source
FY15112	h^{90} *ade6-216 leu1-32 lys1-131 ura4-D18 hht1::hht1-GFP-HA-Kanr*	NBRP, Japan
FY16839	h^{90} *leu1-32 ura4-D18 mcp5::ura4+*	NBRP, Japan
FY16854	h^{+} *his2 leu1-32 ura4-D18 mcp5::[mcp5-GFP-3′UTR-Lys3+]*	NBRP, Japan
FY16887	h^{90} *leu1-32 (mcp5::ura4+)::GFP-mcp5*	NBRP, Japan
FY16897	h^{-} *ade6-M216 ura4-D18 (mcp5::ura4+)::mcp5c-cD*	NBRP, Japan
FY6871	h^{+} *ade6-M210 ura4-D18 leu1*	NBRP, Japan
FY21150	h^{-} *leu1 ura4 dhc1Δ::ura4 (DHC106-1)*	NBRP, Japan
KI001	h^{+} *sid4-GFP::kanr kanr-nmtP3-GFP-atb2+ nmt1-pCOX4RFP::leu1+ ura4-D18 ade6-M210*	Iva Tolić
L972	h^{-} *WT*	Iva Tolić
L975	h^{+} *WT*	Iva Tolić
MM3246	h^{-} *leu1-32 ura4-D18 aco1:GFP:ura4+*	Fuyuki Ishikawa
MTY271	h^{-} *mCherry-atb2:hphMX6 leu1-32 ura-d18*	Masakatsu Takaine
PHP14	h^{-} *ade6-M216, leul-32, ptp-1,* $[rho^{0}]$	Thomas D. Fox
PT1650	h^{+} *cox4-GFP:leu1 ade6-M210 leu1-32 ura4-D18*	Phong Tran
PT1651	h^{-} *cox4-RFP:leu1 ade6-M210 leu1-32 ura4-D18*	Phong Tran
PT2244	h^{+} *mmb1Δ:Kanr cox4-GFP:leu2 mCherry-atb2:Hygr ade6-m210 leu1-32 ura4-d18*	Phong Tran
SV56	h^{90} *dhc1-3xGFP:kan r leu1-32 lys1 ura4-D18*	Iva Tolić
SV91	h^{90} *mcp5-mCherry-kanr dhc1-GFP-Leu2 leu1-32 lys1 ura4-D18*	Iva Tolić
VA019	h^{90} *mCherry-atb2:hphMX6 leu1-32 ura-d18 (mcp5::ura4+)::GFP-mcp5*	This study
VA066	h^{-} *cox4-RFP:leu1 mcp5::ura4+ ade6-M210 leu1-32 ura4-D18*	This study
VA074	h^{+} *cox4-GFP:leu1 mcp5+:: ura4+ ade6-M210 leu1-32 ura4-D18*	This study
VA080	h^{-} *mmb1Δ:Kanr cox4-GFP:leu2 mCherry-atb2:Hygr ade6-m210 ura4-d18*	This study
VA086	h^{+} *cox4-RFP:leu1 ade6-M210 ura4-D18*	This study
VA091	h^{-} *cox4-GFP:leu1 dhc1Δ::ura4 (DHC106-1) ade6-M210 leu1-32*	This study
VA092	h^{+} *cox4-RFP:leu1 dhc1Δ::ura4 (DHC106-1) ade6-M210 leu1-32*	This study
VA099	h^{90} *dhc1-3xGFP:kan cox4-RFP:leu1 ura4-D18*	This study

NBRP, National BioResource Project.

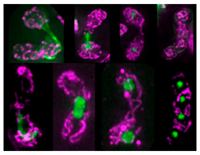

Video 1. **Mitochondrial morphologies during the different stages of meiosis.** Top: 3D projection of microtubules (green) and mitochondria (magenta) in a cross between strains KI001 and PT1651 (see Table S1). Mitochondria appear fragmented and do not associate with microtubules. Bottom: 3D projection of nucleus (green) and mitochondria (magenta) in a cross of strain FY15112 (see Table S1). Mitochondria localize preferentially to the poles of the cell. This video corresponds to Fig. 1, A and B.

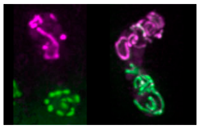

Video 2. **3D projections of GFP-labeled mitochondria (green) and RFP-labeled mitochondria (magenta) in a cross between strains PT1650 and PT1651 (see Table S1).** This video corresponds to Fig. 2 A (horsetail, left; and ascus, right).

https://doi.org/10.1083/jcb.201901108

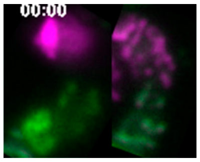

Video 3. **Live-cell confocal microscopy of a cross between strain PT1650 and PT1651 (see Table S1).** This movie corresponds to Fig. 2 C. Time is indicated in hours:minutes. The video is played at 6 frames per second.

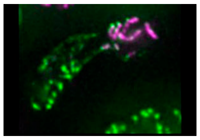

Video 4. **3D projection of Mcp5 (green) and mitochondria (magenta) in a cross between strains FY16854 and PT1651 (see Table S1).** This video corresponds to Fig. 3 A.

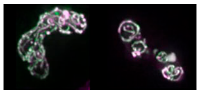

Video 5. **3D projections of GFP-labeled mitochondria (green) and RFP-labeled mitochondria (magenta) in a cross between strains VA066 and VA074 (see Table S1).** This video corresponds to Fig. 3 C (horsetail, left; and ascus, right).

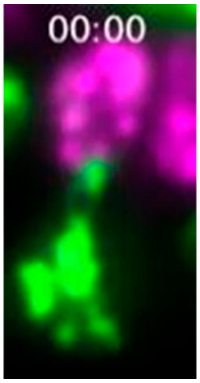

Video 6. **Live-cell confocal microscopy of a cross between strain VA066 and VA074 (see Table S1).** This video corresponds to Fig. 3 E. Time is indicated in hours:minutes. The video is played at 6 frames per second.

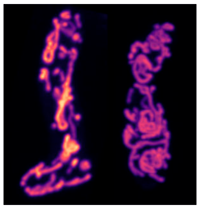

Video 7. **3D projections of mitochondria (warmer colors indicate higher intensities) in a cross between strains FY16897 and PT1650 (see Table S1).** This video corresponds to Fig. 4 A (horsetail, left; and ascus, right).

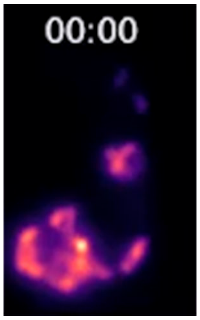

Video 8. **Live-cell confocal microscopy of a cross between strain FY16897 and PT1650 (see Table S1).** This movie corresponds to Fig. 4 C. Time is indicated in hours:minutes. The video is played at 6 frames per second.

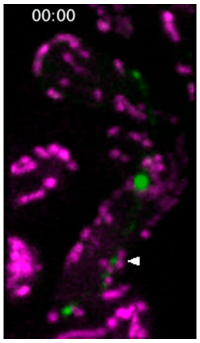

Video 9. **Live-cell spinning disk confocal microscopy of a zygotes from strain VA099 (see Table S1) with fluorescent dynein (green) and mitochondria (magenta).** The arrowhead points to an anchored dynein spot. This video corresponds to Fig. 5 A. Time is indicated in minutes:seconds. The video is played at 1 frame per second.

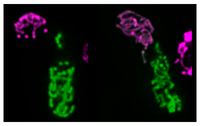

Video 10. **3D projections of GFP-labeled mitochondria (green) and RFP-labeled mitochondria (magenta) in a cross between strains VA091 and VA092 (see Table S1).** This video corresponds to Fig. 5 C (horsetail, left; and ascus, right).
